# Distinct transthyretin oxidation isoform profile in spinal fluid from patients with Alzheimer’s disease and mild cognitive impairment

**DOI:** 10.1186/1559-0275-11-12

**Published:** 2014-03-29

**Authors:** Keld Poulsen, Justyna MC Bahl, Anja H Simonsen, Steen G Hasselbalch, Niels HH Heegaard

**Affiliations:** 1 Department of Clinical Biochemistry, Immunology and Genetics, Statens Serum Institut, Artillerivej 5, DK-2300 Copenhagen S, Denmark; 2 Memory Disorders Research Unit, Department of Neurology, Copenhagen University Hospital, Rigshospitalet, Blegdamsvej 9, DK-2100 Copenhagen Ø, Denmark; 3 Department of Clinical Biochemistry and Pharmacology, Odense University Hospital, University of Southern Denmark, Sdr. Boulevard 29, DK-5000 Odense C, Denmark

**Keywords:** Immunoaffinity, Mass spectrometry, Isoform profiling, Oxidation, Alzheimer’s disease, Transthyretin, Cerebrospinal fluid, Normal pressure hydrocephalus

## Abstract

**Background:**

Transthyretin (TTR), an abundant protein in cerebrospinal fluid (CSF), contains a free, oxidation-prone cysteine residue that gives rise to TTR isoforms. These isoforms may reflect conditions *in vivo*. Since increased oxidative stress has been linked to neurodegenerative disorders such as Alzheimer’s disease (AD) it is of interest to characterize CSF-TTR isoform distribution in AD patients and controls. Here, TTR isoforms are profiled directly from CSF by an optimized immunoaffinity-mass spectrometry method in 76 samples from patients with AD (n = 37), mild cognitive impairment (MCI, n = 17)), and normal pressure hydrocephalus (NPH, n = 15), as well as healthy controls (HC, n = 7). Fractions of three specific oxidative modifications (S-cysteinylation, S-cysteinylglycinylation, and S-glutathionylation) were quantitated relative to the total TTR protein. Results were correlated with diagnostic information and with levels of CSF AD biomarkers tau, phosphorylated tau, and amyloid β_1-42_ peptide.

**Results:**

Preliminary data highlighted the high risk of artifactual TTR modification due to *ex vivo* oxidation and thus the samples for this study were all collected using strict and uniform guidelines. The results show that TTR is significantly more modified on Cys(10) in the AD and MCI groups than in controls (NPH and HC) (p ≤ 0.0012). Furthermore, the NPH group, while having normal TTR isoform distribution, had significantly decreased amyloid β peptide but normal tau values. No obvious correlations between levels of routine CSF biomarkers for AD and the degree of TTR modification were found.

**Conclusions:**

AD and MCI patients display a significantly higher fraction of oxidatively modified TTR in CSF than the control groups of NPH patients and HC. Quantitation of CSF-TTR isoforms thus may provide diagnostic information in patients with dementia symptoms but this should be explored in larger studies including prospective studies of MCI patients. The development of methods for simple, robust, and reproducible inhibition of *in vitro* oxidation during CSF sampling and sample handling is highly warranted. In addition to the diagnostic information the possibility of using TTR as a CSF oxymeter is of potential value in studies monitoring disease activity and developing new drugs for neurodegenerative diseases.

## Introduction

Alzheimer’s disease (AD) is the most common type of dementia. The estimated worldwide prevalence of AD was 36.6 million in 2010 and is estimated to increase to 115 million in 2050 [1]. AD is heterogeneous both at clinical and neuropathological levels [2]. Considerable research efforts are aimed at developing better therapies tailored for the individual patients [3]. For this and for the sake of the patient’s quality of life accurate diagnostic tools are needed, especially in early phases characterized by mild cognitive impairment (MCI) and episodic memory loss that may also be a manifestation of a plethora of other conditions. Today, the AD diagnosis relies on a combination of clinical, imaging, and laboratory criteria [4,5] the latter being cerebrospinal fluid (CSF) levels of amyloid beta 1-42 peptide (Aβ_1-42_), tau protein (t-tau), and tau specifically phosphorylated at Thr-181 (p-tau_181_). The laboratory tests, however, perform less than satisfactory in the early diagnosis of sporadic AD. Thus, many studies focus on developing new biomarkers for AD but no consistently reproducible results about CSF peptide, protein, lipid and metabolite biomarker candidates are available. One potential marker is the most abundant CSF protein transthyretin (TTR). This protein is prone to modifications on its free Cys (10) residue and thus may reflect oxidative stress. Oxidative stress and the presence of reactive oxygen species (ROS) are involved in AD pathogenesis. However, results in the literature are conflicting on TTR concentrations and quantitative proportions of the cysteine oxidations [6-12]. Thus, some studies indicate that TTR concentrations in CSF are altered in AD [13,14] and decrease during AD progression [15-17]. In contrast, CSF-TTR is reported to increase during normal aging [18]. Accordingly, we worked out a method for high-resolution mass spectrometry (MS) of intact TTR where a polyclonal antibody is used to capture all TTR isoforms. Using on-line immunoaffinity (IA) chromatography of crude serum analysed with electrospray ionization (ESI) time-of-flight (TOF) mass spectrometry (MS) the method was initially used to characterize TTR-amyloidosis [19]. The method was modified using immunoprecipitation (IP) of TTR directly from crude CSF to minimize *ex vivo* protein artifacts [20]. The aim of the present study was to investigate the thiol-conjugated TTR isoforms as support for the differential diagnosis of AD and related dementias by applying the method on clinically well-characterized samples collected under uniform and highly controlled conditions. The samples represent AD patients and selected controls (MCI, normal pressure hydrocephalus (NPH) and healthy controls (HC)). The MCI group was a heterogeneous mix of cognitive impaired patients, some later developing AD and others stabilizing at a mild level of cognitive impairment, not reaching AD [21,22]. NPH constitutes an important control group that also may present with symptoms of cognitive impairment. NPH usually develops in individuals past 60 years of age and is due to an abnormal accumulation of CSF in the brain ventricles. Although the cognitive deficiency in NPH is not necessarily associated with disturbances in CSF dynamics, the dementia is potentially reversible by shunt surgery [23]. Unfortunately, NPH is often misdiagnosed as AD, Parkinson’s disease or chronic dementia [24] and better diagnostic tools for this condition are therefore highly needed. The final group of controls in this study constitutes HC that were patients undergoing spinal anaesthesia and had no signs of dementia or cognitive impairment.

## Materials and methods

### Participants

A total of 69 patients with symptoms of dementia and 7 non-demented controls were included in the study (Table 1). The 76 individuals represent four groups: AD, MCI, NPH, and HC. CSF samples and corresponding clinical and paraclinical information were obtained from the Danish Dementia Biobank at the Memory Clinic and Memory Disorders Research Unit (Copenhagen University Hospital, Rigshospitalet, DK). According to the guidelines given by the Ethic committee of the Capital Region of Denmark patients or their closest relatives gave consent for using samples for research purposes.

**Table 1:** Summary of demographic data for samples included in this study

	**AD**	**MCI**	**NPH**	**HC**
n (male/female)	37 (22/15)	17 (12/5)	15 (12/3)	7 (4/3)
Age (years)^a^	71 ± 9	71 ± 9	76 ± 7*	62 ± 12*
Age range (min-max)	45-88	55-89	63-85	51-88

^a^Mean ± SD. The asterisk indicate a significant difference with p < 0.05 between the NPH and HC groups (Mann–Whitney U test).

Global cognitive function was assessed using Mini Mental State Examination (MMSE) [25] and Addenbrooke’s Cognitive Examination (ACE) [26] scores. Diagnoses were based on, as a minimum, neurological examination, cranial CT or MRI, blood and CSF screening tests and cognitive testing. CSF laboratory analyses included quantitative immunoassays for Aβ_1-42_, t-tau and p-tau_181_. The 37 AD patients met the NINCDS-ADRDA criteria for probable AD [27]. The clinical criteria applied for the 17 MCI patients were those defined by Petersen et al. [28]. The 15 NPH patients presented with the triad of gait disturbance, urinary incontinence, and dementia/mental decline. NPH was confirmed by imaging of dilated ventricles in all cases. These criteria correspond to probable NPH [29]. The 7 healthy controls (HC) were patients that underwent minor surgery (*e.g.* hernia repair) under spinal anaesthesia but that were otherwise physically and mentally healthy with no evidence of cognitive decline.

### CSF sampling

CSF was obtained by lumbar puncture between the 3^rd^ and 4^th^ lumbar vertebrae; withdrawing a total of 10–12 mL. Samples were obtained between 10 a.m. and 1 p.m. The first 2 mL were used for routine laboratory analyses. The remaining CSF was collected into a polypropylene tube and was mixed gently to avoid gradient effects and immediately centrifuged at 2000 x *g* for 10 min to precipitate cells and other insoluble material. The supernatant was divided in aliquots of 250 μL in polypropylene cryo-tubes and stored at -80° within 2 hours after sampling. Samples were subsequently sub-aliquotted by quick thawing and gentle mixing of the 250 μL sample, before subdividing into aliquots of 40–50 μl and placing at -80°C. The samples were then kept at -80°C until immunoprecipitation of transthyretin (TTR-IP) was performed.

### Quantitative immunoassays of total transthyretin, Aβ_1-42_, t-tau and p-tau

Aβ_1-42_, t-tau and p-tau_181_ in CSF were quantified using sandwich ELISA kits (Innotest β-Amyloid_(1–42)_, Innotest hTAU-Ag and Innotest Phospho-TAU_(181p)_; Innogenetics, Ghent, Belgium) conducted as described in the vendor protocols and based on previous studies [30,31]. The guidelines used for cut-off values were: Aβ_1-42_ < 550 pg/mL; t-tau > 300 pg/mL (age < 51 years), t-tau 450 pg/mL (age 51–70) and t-tau > 530 pg/mL (age > 70); p-tau_181_ 80 pg/mL. The presence of two abnormal out of the three biomarkers supports the AD diagnosis.

Quantitative rocket immunoelectrophoresis was used to measure total CSF-transthyretin and was carried out as follows: One hundred mL of 1.0% (w/v) agarose (HAS, Litex) in Tris/Tricine electrophoresis buffer was prepared and allowed to cool to 56°C. A volume of 100 μL polyclonal anti human-TTR (Dako A0002, conc. 3.9 mg/ml) was added to 30 mL of agarose solution and poured onto 10×20 cm glass plate (0.5 μL /cm^2^). Wells (3 mm diameter) were punched using a gel puncher with suction, towards one edge of the plate. The wells were placed towards the cathode. TTR purified from human plasma was purchased from Sigma-Aldrich (Prealbumin, P1742). The TTR standard was used to generate a standard curve consisting of five calibrators (50, 25, 12.5, 6.25 and 3.1 μg/ml). All the CSF samples were diluted 1:1 in water and 10 μL of diluted standard and CSF samples were added into the appropriate wells and a constant voltage of 2.5 V/cm was applied overnight. Then, the gel was dried and stained with Coomassie blue. The height of the rocket precipitate was measured from the upper edge of the well to the tip of the rocket, and a standard graph was constructed by plotting the concentration of antigen on Y-axis against the height of the rocket on X-axis. The concentration of transthyretin in the CSF samples was calculated by interpolation from this standard curve. The TTR concentration and TTR isoform characterization (by IA-LC-MS) were in all cases determined after only one thaw-freeze cycle, *i.e.*, after analyzing for the AD biomarker panel.

### Quantitative analysis of TTR isoforms by nanoLC-ESI-MS

Immunoprecipitation of TTR using a polyclonal rabbit anti-human antibody and washing steps was performed as previously described [20] using 40 μL CSF. The lyophilized IP-TTR was solubilized in 20 μl of 30% acetonitrile (ACN), 0.1% formic acid (FA) (v/v) in water, by two minutes sonication in a water bath followed by vigorous vortex mixing. The immunoprecpitate solution was then separated by a biocompatible Ultimate 3000 nanoLC system (Dionex, Thermo Scientific) equipped with an Acclaim PepMap300 C18 trap column (300 μm id x 5 mm cartridge, Dionex) and an Acclaim PepMap300 C18 analytical column (75 μm id × 150 mm long, Dionex) with column oven temperature set to 45°C. The columns were in a column-switching set-up with a ten-port switching valve. Sample (5 μl) was loaded from the autosampler via a 20 μl sample loop onto the trap column with flow rate 30 μl/min (2% ACN and 0.1% FA in water) followed by online pre-concentration and desalting for five minutes. After column-switching the flow rate was 400 nL/min and sample was eluted to the analytical column by (A) 2% ACN and 0.1% FA in water, (B) 95% ACN and 0.1% FA in water: 30% B from 0 to 5 min, 30-100% B from 5 to 59 min (convex gradient 8, Chromeleon software, Dionex). An LTQ Orbitrap XL mass spectrometer (Thermo Scientific) was used to record the protein spectra over the mass range of m/z 800–2000 with 3 microscans per scan with a resolution of 60.000 (full-width at half-maximum peak height) at m/z 400. The sample was introduced through a metal emitter (ES502) mounted on an ES ion source (ES070) (both from Proxeon, Thermo Scientifc, DK). The instrument was operated in positive ESI mode with a spray voltage of 2.0 kV, tube lens voltage of 250 V and capillary temperature of 220°C. The MS parameters were optimized in the range of m/z 800–2000 by infusing TTR purified from human blood (P1742, Sigma-Aldrich). External calibration was performed with the ProteoMass CalMix solution (MSCAL5-1EA, Supelco, Sigma-Aldrich). Raw spectra covering elution of the four main TTR isoforms were summarized and subsequently deconvoluted (i.e. decharged and deisotoped) by the Xtract algorithm within Qual Browser (Xcalibur ver. 2.0.7, Thermo Scientific) using the three most intense charge envelopes as described earlier [20]. The monoisotopic peak heights computed by the Xtract algorithm were used for calculating the relative quantities of the TTR isoforms. To calculate the relative quantities the four monoisotopic peaks were manually selected (in the case of TTR heterozygotes the 4 double peaks were selected) and summed to hundred percent from the individual sample spectra, with subsequent calculation of relative amounts as explained previously [20]. Confirmation of the three oxidative thiol modifications on the TTR-Cys10 was done by reduction with DDT as demonstrated earlier. Reduction diminished the three peaks to baseline with simultaneous increase of the TTR-Cys10 non-modified peak [19]. Validating the chromatographic elution of the TTR isoforms and identifying the PTMs was performed by tandem MS with subsequent analysis using the Prosight PTM v2.0 on-line software [32].

### Statistical analyses

Differences between the four diagnostic groups were evaluated (correcting for multiple comparisons) using the Kruskal-Wallis nonparametric test followed by the post hoc Dunn’s test. The Mann–Whitney U rank sum test evaluated the sum of ranks by pairwise comparisons of diagnostic groups. Correlations were tested by the Spearman’s rank correlation coefficient. Statistical significance was defined as p < 0.05. All statistical analyses were performed in the Prism ver. 5.03 program (GraphPad Software Inc, La Jolla, CA, USA). Multivariate analysis used the Principal Component Analysis (PCA) algorithm implemented in the Latentix v. 2.11 program (http://www.latentix.com). After autoscaling and analysis the PCA scores and loadings plot data were imported into the GraphPad Prism program for graphic presentation.

## Results

### Subjects and samples

Demographic data are summarized in Table 1. The mean ages between the three dementia disease control groups were not significantly different while the HC group was significantly younger than the NPH group (p = 0.011). All CSF samples had an erythrocyte count < 500/μl. The CSF total protein concentration exceeded 0.60 g/L in seven of the 76 patients, four were AD, two were MCI (both with AD as follow-up diagnosis) and one was NPH (follow-up diagnosis: NPH combined with apoplexia).

### CSF biomarkers for AD

**Figure 1:**
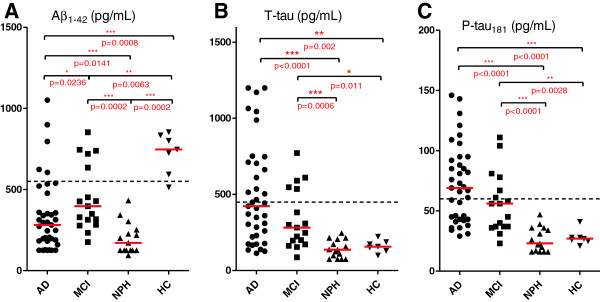
**CSF biomarkers of AD.** CSF Aβ_1-42_**(A)**, t-tau **(B)** and p-tau_181_**(C)** in patients with Alzheimer’s disease (AD), mild cognitive impairment (MCI), normal pressure hydrocephalus (NPH) or healthy controls (HC). Horizontal red bars indicate median values. Horizontal dotted lines indicate recommended cut-off levels for the diagnosis of AD; Aβ_1-42_ < 550 pg/mL, t-tau > 450 pg/mL (age 51–70 years) and p-tau > 60 pg/mL [31]. Statistical significant differences (pairwise analysis by the Mann–Whitney U test) are indicated with horizontal brackets (***, p < 0.001; **, p < 0.01 level; *, p < 0.05). Exact p values are given below each bracket.

The biomarker panel, Aβ_1-42_, t-tau, and p-tau_181_, was measured in all 76 individual CSF samples (Figure 1). Aβ_1-42_, was decreased in all three disease groups as compared to healthy controls with the lowest value in NPH (p = 0.0002), followed by AD (p = 0.0008) and MCI as least significant (p = 0.0063). The order of Aβ_1-42_ concentrations thus was: HC > MCI > AD > NPH (Figure 1A). The lowest level observed was in the NPH group at 201 (125–304) pg/mL compared to AD 281 (183–418) pg/mL, MCI 397 (299–678) pg/mL and the HC group 747 (593–834) pg/mL (median values, 25^th^–75^th^ percentile).

Compared to the healthy controls (median at 157 (130–187) pg/mL) the level of t-tau was significantly elevated as expected in the AD group at 423 (226–708) pg/mL, p = 0.002) and less but still significantly elevated in the MCI group at 282 (185–542) pg/mL (p = 0.011). The NPH group at 137 (75–207) pg/mL was not significantly different from the HC group. The order of t-tau concentrations thus were: AD > MCI > HC > NPH (Figure 1B). The p-tau_181_ concentrations follow the same rank as the t-tau concentrations (Figure 1C). The median value for p-tau_181_ in HC was 27 (25–28) pg/mL compared to AD at 69 (43–94) pg/mL (p < 0.0001) and MCI at 56 (37–73) pg/mL (p = 0.0028). Again, the NPH group at 23 (16–36) pg/mL is not statistically different from the HC group.

### Sample handling for immunoprecipitation (IP)-mass spectrometric TTR isoform profiling

**Figure 2:**
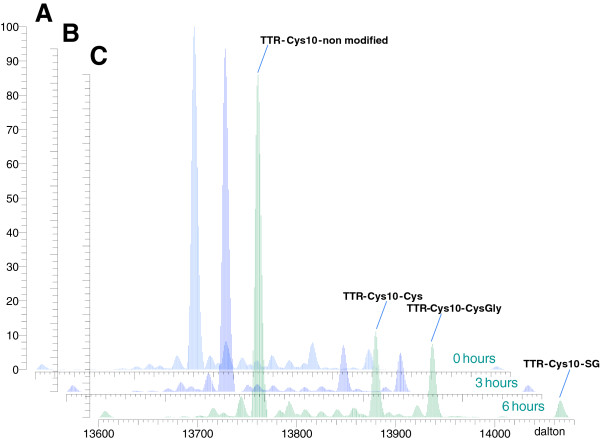
**Storage time influences TTR oxidation profiles.** Deconvoluted spectra of IP-TTR, 0 hours at 4°C before IP **(A)**, IP-TTR, 3 hours at 4°C before IP **(B)** and IP-TTR, 6 hours at 4°C before IP **(C)**.

Preliminary experiments [20] showed the possibility of using IP-MS to quantitate TTR oxidation isoforms directly from crude CSF. Before using the approach for analysing the cohort of samples included in the present study, conditions were worked out to minimize artifactual oxidation. Very pronounced *in vitro* effects were seen if samples were kept at room temperature or even at 4°C for few hours. Thus, Figure 2 shows deconvoluted and superimposed spectra from three aliquots of the same sample sample kept at 4°C for 0, 3 or 6 hours (300 rpm on horizontal shaker). The peak-heights of TTR-Cys10-Cys (cysteinylated), TTR-Cys10-CysGly (cysteine-glycinylated), and TTR-Cys10-SG (glutathionylated) increase proportionally with storage time. These results clearly document the need for controlled sample handling. Therefore, to keep handling artifacts at a minimum the study samples were all collected uniformly and with as short a handling time and as few thaw-freeze cycles as practically possible (*cf.* Materials and methods).

### Optimization and data handling on NanoLC ESI Orbitrap MS of intact TTR isoforms

**Figure 3:**
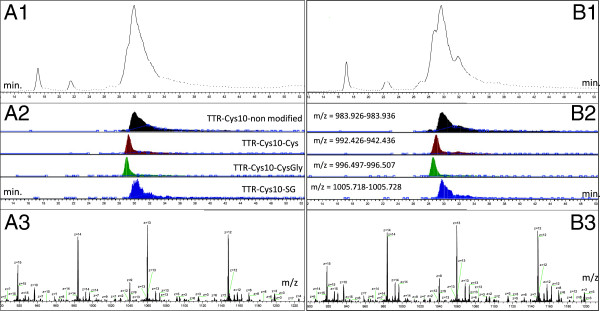
**TTR isoform data extraction.** Chromatographic elution profiles from the 3 hour (A) and the 6 hour (B) samples (from Figure 2). The TTR isoforms elute with substantial overlap **(A1 and B1)**, from 28 to 34 min. In the panels below **(A2 and B2)** extracted chromatograms are presented for all four TTR isoforms and differential peak elution times can be observed. For peak extractions the theoretical isoform masses were calculated and the m/z windows were manually adjusted according to the precision of the external calibration. The calibration was minor, routinely 0–3 ppm. Spectra across the four elution peaks were combined **(A3 and B3)**. These raw spectra were used for deconvolution and subsequent semi-quantitative calculations as described in Materials and Methods.

Data extracted from the chromatographic elution profiles of the 3 hour (A) and the 6 hour (B) samples (from Figure 2) are presented in Figure 3. It is observed (Figure 2A1 and B1) that the TTR isoforms elute with substantial overlap, almost as one peak. In the extracted chromatograms (A2 and B2) of all four TTR isoforms differential peak elution can be observed. For peak extractions the theoretical masses of the four isoforms were calculated using manual adjustment of the m/z windows according to the precision of the external calibration. The calibration was routinely on the scale of 0–3 ppm and only minor adjustments were used. The extraction windows were: TTR-Cys10 non modified, m/z = 983.926-983.936; TTR-Cys10-Cys, m/z = 992.426-992.436; TTR-Cys10-CysGly, m/z = 996.497-996.507 and TTR-Cys10-SG, m/z = 1005.718-1005.728. In A3 and B3 the summarized raw spectra across the four elution peaks are shown. These spectra were used for deconvolution and subsequent semi-quantitative calculations as described in Materials and methods. In the previously published method direct infusion of immunoprecipitated sample to the MS was used [20] but we find that the infusion flow by itself has a major impact on the relative abundance of the spectral peaks (data not shown) (metal emitters have been reported to cause unwanted sample oxidation [33-35]) and we here instead use reversed phase separation in line with the mass spectrometer.

### TTR Cys-10 oxidative isoforms

**Figure 4:**
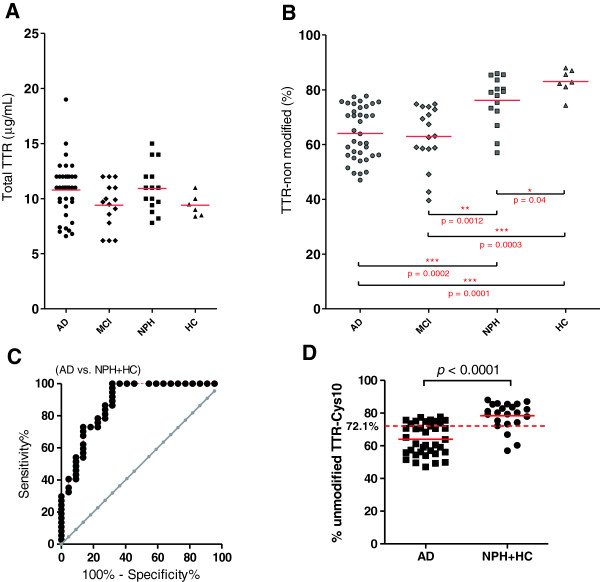
**Altered CSF-TTR isoform profile in AD.** Data show CSF total transthyretin (TTR) **(A)** and the fraction of non-modified TTR **(B)** in patients with Alzheimer’s disease (AD), mild cognitive impairment (MCI), normal pressure hydrocephalus (NPH) or healthy controls (HC). Horizontal red bars indicate median values. **(C)** Receiver operating characteristics (ROC) curve. Alzheimer’s disease (AD) (n = 39) were compared HC and NPH controls (n = 22). Area under curve (AUC) was 0.88 with p-value <0.0001, optimal cut-off is 72.1% non-modified TTR. **(D)** Levels of % non-modified TTR in CSF from patients with AD and the combined control group of NPH and HC. Statistical significant differences (pairwise analysis by the Mann–Whitney U test) are indicated with horizontal brackets (***, p < 0.001; **, p < 0.01 level; *, p < 0.05).

After analyzing all samples by IA-LC-MS as described data were expressed as the fraction of unmodified TTR relatively to the sum of all TTR peak heights in each sample (Figure 4). When analyzing the same sample repeatedly the intra-assay variability of this parameter was up to about 7% (data not shown). Total TTR concentration was between 6.2 and 19.0 μg/mL (overall mean ± 1 SD = 10.1 ± 1.9 μg/mL) with no significant differences in TTR concentrations between any of the four groups (Figure 4A). CSF total protein and total TTR were also tested independently for correlations with patient age using Spearman’s rank correlation coefficient test in all four diagnostic groups and none were found (data not shown). The relative abundance of modified TTR in the AD and MCI group was tested for correlation with gender and none was found.

The TTR isoform results (Figure 4B) showed a clear segregation of the AD and MCI groups from the HC group and to a lesser extent from the NPH group. Also the NPH and HC groups differed slightly (*p* = 0.041). Thus, TTR was significantly more oxidatively modified in AD patients compared to the HC group (p = 0.0001) and compared to the NPH group (p = 0.0002). Likewise, the relative amount of the fraction of modified TTR in MCI patients differed significantly from the HC group (p = 0.0003) and to lesser extend from the NPH group (p = 0.0012).

The use of the routine CSF-biomarker AD panel alone with the usual criteria (2 abnormal out of 3 support the AD diagnosis) gave a diagnostic sensitivity of 57% for AD and a specificity of AD versus the grouping of NPH and HC together, of 95%. The TTR oxidation data were also evaluated for diagnostic performance (AD *vs.* NPH + HC) (Figure 4C and D). A cut-off of 72.1% non-modified TTR was found to be optimal in this cohort yielding a sensitivity and specificity of 73% and 86%, respectively with a value of 0.88 for the area under the ROC curve (p < 0.0001) (Figure 4C).

### Multivariate analysis

**Figure 5:**
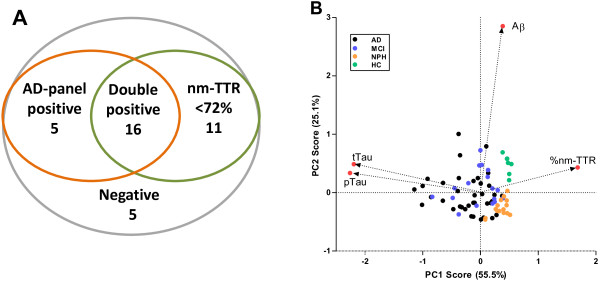
**Multivariate data analysis. A**, Venn diagram illustrating the combination of AD-panel results and % TTR modified results. From 37 patients diagnosed with probable AD, 32 have abnormal AD panel outcome and/or abnormal values for non-modified TTR (nm-TTR). Together, this gives a sensitivity of 86% and a specificity of 82% of the AD diagnosis versus the NPH and HC groups; **B**. scores and loadings plot of the principal components analysis (PCA) using Aβ_1-42_ (Aβ), t-tau (tTau), and p-tau_181_ (pTau) and the % fraction of non-modified TTR (nm-TTR) as input data in the Latentix v 2.11 program. Loading vectors are illustrated by arrows and red dots. Plot data were imported into GraphPad Prism for the graphic presentation.

When combining the AD biomarker panel with the TTR isoform analysis requiring 2 out of 3 parameters of the AD-panel to be abnormal or the amount of non-modified TTR to be below <72 for the AD diagnosis, the diagnostic sensitivity for AD versus the NPH and HC groups was improved to 86% at a specificity of 82%. The Venn diagram (Figure 5A) illustrates how the 37 AD patients score when combining the two tests. The relative contribution of the 4 parameters (amyloid β_1–42_, tTau, pTau, and % non-modified TTR (nm-TTR)) was assessed by principal components analysis (PCA) (Figure 5B. The analysis maximizes the data variability and projects this information into a two-dimensional subspace between samples (the prinicpal components (PC) PC1-PC2 score plot) and provide loading vectors that show how variables and the subspace dimensions relate (Figure 5B). The PCA clearly showed the ability of the data to completely separate HC (green dots, Figure 5B) from the AD/MCI group, the inability to differentiate between AD and MCI, and the pronounced and differential clustering of NPH samples (orange dots, Figure 5B) in a group apart from both the HC and the AD/MCI groups. The loading vectors illustrate the comparable contribution to sample differentiation of t-tau and p-tau and the independent contribution of Aβ_1-42_ (situated to the upper right quadrant because high values are associated with non-AD controls). Also, the plot illustrates the direction of the contribution of the non-oxidized TTR fraction parameter (nm-TTR) that negatively correlates with pTau and tTau. Finally, Figure 5B shows that a selective decrease of Aβ_1-42_ in the presence of normal values of tau species and almost normal values of oxidized-TTR fraction is highly specific for NPH cases in this sample cohort.

## Discussion and conclusions

The main novel finding in the present study is that the abundance of oxidized TTR isoforms is significantly increased in CSF from AD and MCI patients as compared to the HC and the NPH groups. Samples from all patient and control groups were collected under stringent and uniform conditions designed to minimize artifactual oxidation. We cannot confirm a finding [36] of decreased levels of total TTR in CSF from AD and NPH patients. Also, our findings of increased oxidation of TTR in CSF from AD patients is in conflict with earlier reports using linear MALDI-TOF MS for quantitation of TTR isoforms where less TTR-oxidation was found [9]. The previous study, however, analyzed directly on crude CSF dried down with matrix on target and overall find much higher oxidation of TTR, especially in control samples, than in the present study. Also, the spectra are less well resolved and proteins less confidently identified. Thus, method differences, the contribution from other proteins and their isoforms (*e.g.* cystatin C) in the specific m/z-range, and the contribution from *ex vivo* oxidation caused by differences in sample handling may all underlie the discrepancy between the present work and the previous report.

In the present study we furthermore incidentally confirm that the NPH group exhibits a unique biomarker profile with significantly more reduced Aβ_1-42_ values than AD but normal tau levels as previously documented [36-38]. Others have observed a similar reduction of Aβ_1-42_ in NPH but with concurrent reduction of the levels of the two tau species [39,40]. Our data indicate that a larger prospective study of the diagnostic use of quantitation of oxidative TTR isoforms in patients presenting with symptoms of dementia together with standard biomarkers is warranted and that more simple rapid methods, *e.g.* based on immunochemical reagents specific for the non-modified Cys-10 epitope should be developed.

## Competing interests

The authors declare that they have no competing interests.

## Authors’ contributions

KP, JMCB, NHHH conceived the study and designed the experiments; AHS and SH were responsible for sample collection and clinical data; KP carried out the experiments; KP, JMCB, SH, NHHH analyzed the data; KP and NHH wrote the manuscript; JMCB, AHS, SH, NHHH provided critical comments. All authors read and approved the final manuscript.
